# In vitro chemoresponse in metachronous pairs of gyneclologic cancers

**DOI:** 10.1186/2053-6844-1-7

**Published:** 2014-12-06

**Authors:** Heather J Dalton, James Fiorica, Candace K McClure, Rodney P Rocconi, Fernando O Recio, John L Levocchio, Matthew O Burrell, Bradley J Monk

**Affiliations:** The University of Texas MD Anderson Cancer Center, Houston, TX USA; Sarasota Memorial Hospital, Sarasota, FL USA; Precision Therapeutics, Inc, Pittsburgh, PA USA; University of South Alabama Mitchell Cancer Institute, Mobile, AL USA; South Florida Center for Gynecologic Oncology, Boca Raton, FL USA; North Shore LIJ Health System, Biomedical Research Alliance of New York, Manhassett, NY USA; Georgia Gynecologic Oncology, Atlanta, GA USA; University of Arizona Cancer Center, Creighton University School of Medicine at Dignity Health St. Joseph’s Hospital and Medical Center, 500 W. Thomas Road, Suite 600, Phoenix, AZ 85013 USA

**Keywords:** Gynecologic cancer, Drug resistance, Recurrent ovarian, Chemotherapy, Cross-resistance

## Abstract

**Background:**

While most gynecologic cancers respond to first-line cytotoxic chemotherapy, treatment of recurrent disease is frequently associated with acquired drug resistance. In order to find an in vitro surrogate of this clinical phenomenon, a tumor chemoresponse assay was studied.

**Methods/Materials:**

Patients who had tissue submitted for repeated chemoresponse testing were identified through a retrospective search. Sixty-three patients met inclusion criteria (chemoresponse testing completed at primary diagnosis and upon recurrence of disease and assays completed ≥90 days apart). The Wilcoxon signed-rank test was used to compare chemoresponse, represented as a response index (RI), between primary and recurrent measurements. In a secondary analysis, response was categorized and coded as Responsive = 3, Intermediately Responsive = 2 and Non-Responsive = 1, and the paired *t*-test was used to compare chemoresponse between primary and recurrent measurement.

**Results:**

Median time between primary and recurrent tumor testing was 309 days (IQR 208–422). Drugs tested included carboplatin, cisplatin, docetaxel, doxorubicin, gemcitabine, paclitaxel, topotecan, and combination carboplatin/gemcitabine and carboplatin/paclitaxel. There were no differences in chemoresponse between primary and recurrent measurement when chemoresponse was represented by RI scores; although a trend toward increased resistance to paclitaxel upon recurrence was noted. When chemoresponse was analyzed as a continuous variable corresponding to categorized response, a significant shift toward increased resistance to paclitaxel at recurrence, and a marginally significant trend toward increased resistance to carboplatin at recurrence, were observed.

**Conclusions:**

We observed a trend toward increased chemoresistance at recurrence for paclitaxel, and a marginally significant trend toward increased chemoresistance to carboplatin, but no change in chemoresponsiveness between primary diagnosis and recurrence of disease for other common chemotherapy drugs, including common second-line agents such as doxorubicin, gemcitabine, and topotecan.

## Background

It is estimated that in 2014, there were 21,980 new cases of ovarian cancer with 14,270 deaths [1]. The majority of patients present with advanced stage disease. While the majority of women will achieve complete clinical remission after cytoreductive surgery and platinum-based chemotherapy, approximately 30% of ovarian cancer patients do not have a complete response to front-line platinum-based treatment [2–6]. Furthermore, the majority of ovarian cancer patients recur and response rates to second-line treatments are substantially lower. This may be due, in part, to acquired drug resistance [7]. In the recurrent population, empiric-based chemotherapy is associated with response rates ranging from only 5 to 20% with limited progression-free (PFS) and overall survival (OS) [8–11].

Since recurrence rates for ovarian cancer are high and the associated toxicity to chemotherapy can be significant, knowledge of potential tumor responses to chemotherapy a priori could be a useful tool in the selection of effective chemotherapy for patients with advanced or recurrent disease. Ineffective chemotherapy results in unnecessary toxicity and costs, delay of more effective treatment, and the potential development of acquired drug- and cross drug-resistance. An in vitro assay performed before therapy initiation to identify the drug(s) most likely to be effective for the individual patient would have clinical utility. Information provided by an in vitro assay, when integrated with clinical judgment, could lead to the identification of a potentially more effective treatment, thus eliminating toxicity due to ineffective treatments, avoiding a delay in the implementation of effective treatments, and potentially reducing treatment costs. Chemosensitivity and resistance assays are currently recognized in the National Comprehensive Cancer Network (NCCN®) Clinical Practice Guidelines for Oncology for ovarian, fallopian tube, and peritoneal cancers [12, 13]. ChemoFx is a live cell platform-based drug response marker that was developed to overcome the technical limitations of earlier generations of chemotherapy sensitivity and resistance assays and to determine chemosensitivity as well as chemoresistance [14]. Studies of this assay in ovarian cancer indicate that ChemoFx results are predictive of PFS [15] and OS [16].

There is currently limited information on whether chemosensitivity changes throughout the course of adjuvant chemotherapy administration and upon recurrence of disease. Therefore, the primary objective of this study is to determine whether in vitro tumor response differs in metachronous gynecologic cancer specimens collected from the same patient upon primary diagnosis and upon recurrence.

## Methods

### Participants

Inclusion criteria were as follows: (1) tumor samples were submitted to Precision Therapeutics, Inc. (Pittsburgh, Pa) for ChemoFx testing between August 2, 2006 and May 31, 2010; (2) chemoresponse testing was completed at primary diagnosis and upon recurrence of disease; and (3) assays were completed at least 90 days apart. A total of 63 participants met the inclusion criteria. Thirty-six of these patients were enrolled in the ChemoFx Physician Reported Outcomes (ChemoFx PRO) study. The research protocol was reviewed by the Copernicus Group Institutional Review Board, and they determined that this study qualifies as exempt research under 45 CFR §46.101 (b) (4) before the initiation of the analysis. Existing de-identified (anonymized) clinical and pathological data were used to perform the analysis.

### ChemoFx

Fresh tumor specimens preserved in McCoy’s medium were received by the commercial laboratory, usually within 24 hours of removal. Tumor testing methods were reported previously [15]. Briefly, tumor specimens were mechanically disrupted to release and establish malignant epithelial cells as monolayer cultures. The tumor sample was then tested against a series of 10 serial dilutions of each drug or drug combination. The range of drug concentrations tested for each drug(s) was as follows: carboplatin (1 μM-500 μM), cisplatin (0.2 μM-100 μM), docetaxel (0.1 nM-25 nM), doxorubicin (2 nM-1 μM), etoposide (20 nM- 2 μM), gemcitabine (1 nM- 50 nM), ifosfamide (0.2 μM- 100 μM), paclitaxel (0.2 nM-0.1 μM), and topotecan (0.4 nM- 0.2 μM), in addition to combination carboplatin/gemcitabine and carboplatin/paclitaxel. Combination drugs were tested as a 1:1 combination of each drug at each dose, for example, dose 1 of carboplatin-paclitaxel is dose 1 (1 μM) of carboplatin plus dose 1 (0.2 nM) of paclitaxel. After an incubation period of 72 hours, the cells were fixed with anhydrous ethanol (95% fixing grade) and stained with 4’6-diamidino-2-phenylindole (DAPI), a fluorescent DNA stain for imaging the nucleus (Sigma-Aldrich Corp, St Louis, MO, USA). Cells that remained attached after staining were imaged and counted using automated cell microscopy and cell-counting software. The percentages of cells remaining after drug treatment were used to determine survival fraction (SF = average cell count_dosex_/average cell count_control_), from which dose–response curves were plotted. Each dose–response curve was assigned a response index (RI) score ranging from 0 to 10. The RI score is a metric based on adjusted areas under the curve (aAUC); greater RI values represent greater sensitivity. Curves were smoothed using a logarithmic curve-fit tool (GraphPad Prism®, LaJolla, CA, USA). Based on the aAUC score, in vitro tumor response was then categorized into three groups: responsive (R), intermediately responsive (IR), or non-responsive (NR). The thresholds for these classifications were established based on the 25th and 75th aAUC percentiles. Drugs tested included carboplatin, cisplatin, docetaxel, doxorubicin, gemcitabine, paclitaxel, and topotecan, in addition to combination carboplatin/gemcitabine and carboplatin/paclitaxel.

### Statistical methods

The Wilcoxon signed rank test was used to compare chemoresponse (RI score) between primary and recurrent measurement. In commercial reports, tumors are categorized as R, IR or NR to chemotherapies; we therefore completed a second analysis with code corresponding to R = 3, IR = 2 and NR =1, and utilized a two-tailed Wilcoxon signed rank test to compare chemoresponse between primary and recurrent measurement. Statistical significance was set at p < 0.05. All analyses were performed with SAS (version 9.2; SAS Institute, Cary, NC).

## Results

The demographic characteristics of the 63 patients who met inclusion criteria are shown in Table 1. Of the 63 pairs, 44 (70%) were diagnosed with ovarian cancer, 6 (10%) with peritoneal cancer, 4 (6%) with fallopian tube cancer, and 9 (14%) with uterine cancer. Mean age of patients was 59 (±11) years. The majority of cases exhibited poor tumor grade (grade 3) and FIGO Stage III. In the subset of patients with known treatment information (those enrolled in the ChemoFx PRO study), all were treated with a platinum/taxane combination for at least one cycle between in vitro chemoresponse testing of their metachronous tumors. Treatment information in this subsample is shown in Table 2.

**Table 1:** **Characteristics of patients at diagnosis (N = 63)**

**Age (years)**	59.8 ± 11
**Cancer Type**	
Fallopian Tube	4 (6%)
Ovarian	44 (70%)
Peritoneal	6 (10%)
Uterine	9 (14%)
**Tumor Grade**	
G1 Well	1 (2%)
G2 Moderate	6 (10%)
G3 Poor	24 (38%)
G4 Undifferentiated	10 (16%)
Unknown	22 (35%)
**FIGO Stage**	
I	4 (6%)
II	4 (6%)
III	47 (75%)
IV	7 (11)
Unknown	1 (2%)

**Table 2:** **Treatment administered to the subset of patients enrolled in the ChemoFx (Precision Therapeutics, Inc., Pittsburgh, PA) Physician Reported Outcomes Study between assays**

	N = 36
Bevacizumab/Carboplatin/Paclitaxel	2 (6%)
Carboplatin/Cisplatin/Paclitaxel	7 (19%)
Carboplatin/Gemcitabine/Paclitaxel	1 (3%)
Carboplatin/Docetaxel	2 (6%)
Carboplatin/Paclitaxel	22 (61%)
Cisplatin/Docetaxel/Paclitaxel	1 (3%)
Cisplatin/Paclitaxel	1 (3%)

Because specific individual chemotherapy agents or combinations of agents tested in the assay were selected by the treating physician, determination of chemoresponse was not possible for every drug in each patient. Overall, median time between primary and recurrent assay testing was 309 days (IQR = 208,422; minimum 91; maximum 680).

When examined using the RI score, no significant differences were observed in chemoresponse between primary and recurrent assay results (Table 3); however, a trend toward increased resistance was observed for the drug paclitaxel (p = 0.08). When chemoresponse was examined using the commercially reported categories, a significant shift toward chemoresistance were observed for the drug paclitaxel (p = 0.04) and a marginally significant shift was observed for the drug carboplatin (p = 0.06) (Table 4). There were no statistically significant differences between primary and recurrent measurement in the remaining drugs tested.

**Table 3:** **Chemoresponse assay results (expressed as a continuous variable (RI score)) for metachronous tumor pairs**

	Number of pairs	Primary median (IQR) RI SCORE	Recurrent median (IQR) RI SCORE	p
Carboplatin	46	5.59(4.89,6.09)	5.32(4.94,5.80)	0.28
Carboplatin/Gemcitabine	35	5.71(5.16,6.30)	5.90(5.40,6.36)	0.33
Carboplatin/Paclitaxel	41	6.17(5.15,6.67)	5.92(5.25,6.29)	0.48
Cisplatin	42	5.40(4.66,5.77)	5.38(4.67,5.68)	0.80
Docetaxel	42	5.26(4.74,5.75)	4.96(4.43,5.42)	0.14
Doxorubicin	49	5.17(4.20,5.58)	5.11(4.56,5.56)	0.82
Etoposide	39	5.49(4.66,6.00)	5.41(4.83,5.84)	0.85
Gemcitabine	48	5.27(4.46,5.55)	5.11(4.68,5.55)	0.64
Ifosfamide	30	5.58(5.32,5.80)	5.68(5.25,5.87)	0.90
Paclitaxel	47	5.59(4.99,6.21)	5.30(4.80,5.74)	0.08
Topotecan	52	5.39(4.65,5.74)	5.20(4.54,5.65)	0.99

**Table 4:** **Chemoresponse assay results (expressed as a continuous variable corresponding to categorized results (R = 3, IR = 2, NR = 1)) for metachronous tumor pairs**

	Number of pairs	Primary median (IQR) RI SCORE	Recurrent median (IQR) RI SCORE	p
Carboplatin	46	2(1–3)	1(1–2)	0.06
Carboplatin/Gemcitabine	35	2(1–3)	2(2–3)	0.36
Carboplatin/Paclitaxel	41	3(1–3)	2(1–3)	0.67
Cisplatin	42	2(1–2)	2(1–2)	1.00
Docetaxel	42	1(1–2)	1(1–2)	0.51
Doxorubicin	49	1(1–2)	1(1–2)	0.89
Etoposide	39	2(1–2)	2(1–2)	0.21
Gemcitabine	48	1(1–2)	1(1–2)	0.33
Ifosfamide	30	2(1–2)	2(1–2)	0.83
Paclitaxel	47	2(1–3)	1(1–2)	0.04
Topotecan	52	1.5(1–2)	1(1–2)	0.70

## Discussion

Our results suggest that chemoresponsiveness does not change between primary diagnosis of disease and recurrence of disease for the majority of drugs in patients with gynecologic cancer who experienced recurrence within approximately one year. These results are consistent with a large analysis of 334 metachronous pairs of epithelial ovarian cancer specimens performed by Tewari et al. Their analysis failed to show a significant difference in the drug resistance profile of primary tumors and matched recurrences in the same patient [17]. These findings, in addition to ours, may be unexpected but not inexplicable. Furthermore, these results are consistent with observations of biomarker expression in metachronous pairs of epithelial ovarian cancer. No significant changes in the expression of MDR1, p53, or HER2 were noted between primary diagnosis and relapse of disease in 66 patients [18].

A possible explanation for continued chemosensitivity following exposure to chemotherapy, as observed in our primary analysis, has been offered by Tewari et al. They postulate that after initial debulking surgery, patients may harbor residual tumor in poorly vascularized areas. Such tumors would be shielded from systemic chemotherapy, retain their initial drug responsiveness, and contribute to tumor regrowth and recurrence. Because the length of time between end of treatment and subsequent recurrence plays an important role in the development of drug resistance, [19] it is possible that if we had examined patients with longer time to recurrence, we may have observed greater shifts toward increased resistance.

A study conducted by Zajchowski, et al. examined differences in biomarker expression via immunohistochemical analysis in primary and recurrent ovarian cancer specimens over a much longer time interval. In the majority of patients examined, the interval between treatments was >2 years, and as high as ≈ 8 year from the primary to last-received recurrent specimen. These patients received between 1–5 prior chemotherapies. Though differences were found in the expression of certain markers within each matched tumor pair, overall biomarker profiles for the matched patient specimens were very similar [20]. A study examining a larger patient population is needed to validate these findings.

A commonly held view is that chemoresponsiveness may change between primary diagnosis and recurrence of disease with intervening administration of chemotherapy. This may be due to the selection and clonal expansion of drug-resistant cells. Indeed, we observed a trend toward increased resistance to paclitaxel in our primary analysis, and a significant shift toward increased chemoresistance for paclitaxel, and a marginally significant shift toward increased chemoresistance for carboplatin, when examining change categorically. Although the treatment information was not included in the scope of this entire analysis, many of the patients in this study are assumed to have received platinum/taxane-based treatment as first-line chemotherapy. This assumption is supported by the treatment information garnered from the subset of patients enrolled in the ChemoFx PRO study. Exposure to these particular drugs may, in part, explain the more evident shift towards resistance observed with carboplatin and paclitaxel from primary to recurrent tumor (i.e., acquired drug resistance). Notably, there was no evidence for the development of cross-resistance; we did not observe an increase in resistance at recurrence for common second-line treatment agents, such as doxorubicin, gemcitabine, and topotecan.

The theory of acquired drug resistance may explain our results and those of a previous study of metachronous pairs conducted by Matsuo et al.; paclitaxel resistance was higher upon recurrence in a sample of 29 epithelial ovarian cancer patients treated with adjuvant platinum/taxane therapy. In addition, in a sample of 65 patients, recurrent surgery after initial cytoreduction was significantly associated with increased resistance to paclitaxel [19]. Similar increases in paclitaxel resistance in recurrent disease have been reported in other series [20, 21]. Additional studies have shown limited, albeit, significant increases in carboplatin [22] and more commonly, paclitaxel, [22, 23] chemoresistance throughout the course of disease in primary epithelial ovarian cancer, with no changes in any other cytotoxic agents examined. Clinically, it has been shown that while taxanes have activity as later-line agents in ovarian cancer, resistance emerges over time and this increased resistance is very likely due to intervening therapies [24].

Heterogeneity of drug response is a serious clinical problem encountered when administering chemotherapy. Chemoresponse assays may help the physician avoid administering certain chemotherapeutic agents when a patient’s tumor is found to be resistant to those agents. Our results suggest that the chemosensitivity profiles provided by ChemoFx in the primary setting may be valuable for use in both the primary and recurrent setting. Since very few patients undergo a secondary surgery, the opportunity to use results from tissue collected at initial cytoreduction underscores the importance of our current findings. The primary setting may be the physician’s only chance to collect a specimen and thus chemoresponse results in the primary setting may be critical to that patient’s future treatment.

The limitations of this study are those inherent to retrospective studies, including potential selection biases and lack of blinding and control groups. We were also limited in statistical power by a small sample size and short time to recurrence. Additionally, the use of assay results to guide chemotherapy treatment may be a potential confounder.

## Conclusions

In summary, we observed a trend toward increased chemoresistance at recurrence for the common first-line gynecologic cancer treatment agent paclitaxel, and a marginally significant trend toward increased chemoresistance at recurrence for the first-line gynecologic cancer treatment agent carboplatin, but no change in chemoresponsiveness between primary diagnosis and recurrence of disease for other common chemotherapy drugs, including common second-line agents such as doxorubicin, gemcitabine, and topotecan. Our results are consistent with clinical observations and established biologic concepts, suggesting that an in vitro assay may be a useful tool in both research and clinical practice. Drug resistance remains a major obstacle in cancer therapy. Further research is needed to better elucidate the mechanisms by which tumors acquire drug resistance and to define the role of chemoresistance assays in the treatment of gynecologic cancers.
